# Association of rs712 polymorphism in a let-7 microRNA-binding site of *KRAS* gene with colorectal cancer in a Mexican population 

**DOI:** 10.22038/ijbms.2019.26564.6507

**Published:** 2019-03

**Authors:** Martha Patricia Gallegos-Arreola, Guillermo Moisés Zúñiga-González, Karen Gómez-Mariscal, Mónica Alejandra Rosales-Reynoso, Luis Luis, Ana María Puebla-Pérez, Tomas Pineda-Razo

**Affiliations:** 1 División de Genética, Centro de Investigación Biomédica de Occidente, Instituto Mexicano del Seguro Social, Guadalajara, Jalisco, México; 2 Medicina Molecular, Centro de Investigación Biomédica de Occidente, Instituto Mexicano del Seguro Social, Guadalajara, Jalisco, Mexico; 3 Laboratorio de Inmunofarmacología, Centro Universitario de Ciencias Exactas e Ingenierías, Universidad de Guadalajara, Guadalajara, Jalisco, Mexico; 4 Servicio de Oncología, Unidad Médica de Alta Especialidad, Hospital de Especialidades, Centro Médico Nacional de Occidente, Instituto Mexicano del Seguro Social, Guadalajara, Jalisco, Mexico

**Keywords:** Colorectal cancer, KRAS, let-7, Mexican population, Polymorphism

## Abstract

**Objective(s):**

The rs712 polymorphism in a let-7 microRNA-binding site at *KRAS* gene has been associated with cancer. To examine its association with rs712 polymorphism, we analyzed Mexican individuals with colorectal cancer (CRC) and healthy subjects.

**Materials and Methods:**

Genotyping of the rs712 polymorphism was performed by polymerase chain reaction in 281 controls and 336 CRC patients.

**Results:**

The observed frequencies of rs712 polymorphism indicated an associated protective factor for CRC (*P*=0.032). An association between genotype and the disease was evident in: colon localization (allele *T,* odds ratio (OR) 3.82, 95% confidence Intervals (CI) 2.77-5.28, *P*=0.0001), node metastasis (genotype* TT,* OR 2.49, 95% CI 1.45-4.28, *P*=0.0009), poor differentiation (genotype GT, OR 2.35, 95% CI 1.35-4.1, *P*=0.0033), and poor chemotherapy response (genotype GT, OR 2.6, 95% CI 1.7-4.24, *P*=0.0001).

**Conclusion:**

Comparison of the data from patients with control group showed that polymorphism of rs712 in *KRAS* gene was protective factor, which was associated with susceptibility for CRC. However, the genotypes *TT* and *GT* of rs712 polymorphism in *KRAS* could contribute significantly to colon localization, node metastasis, poor differentiation and poor chemotherapy response in CRC patients in this sample population.

## Introduction

Colorectal cancer (CRC) is a serious public health problem in Mexico and the world (1-3), and its incidence varies between different ethnic groups (2-5). In Mexico, CRC is associated with 4% of cancer dead (6). CRC is thought to develop through a gradual accumulation of genetic changes that could modify the intestinal cells (1, 3). In this sense, many studies have shown the relationship of *KRAS* gene with CRC, so that the K-Ras protein (first identified in Kirsten rat sarcoma virus) is a part of RAS/MAPK signaling pathway, and their function is through GTPase that acts like switch by converting the active GTP molecule to inactive GDP, which is essential in the cellular signal process that control the growth, maturation and cellular death (7). Two copies of the *KRAS* gene exist, the pseudogene *KRAS1* and *KRAS2* gene, which are localized at 12q12.1 chromosome, and contain 6 exons of which the exons 1, 5 and 6 are non-coding, and 4 exon join by alternative splicing to make 2 mRNA transcripts known as isoforms 4A (active) and 4B (inactive) (7, 8). The KRAS protein is a proto-oncogene, which is regulated by proteins that bind to the promoter regions of the gene in the initiation of transcription by microRNAs (miRNAs) molecules that act on the elongation phase of transcription. The miRNAs regulate gene expression in the 3’UTR region of mRNA. These interactions between microRNA and mRNA destabilize the mRNA and repress protein synthesis. MicroRNAs such as *let-7*, *lin-4* and bentam, regulate proliferation and differentiation of the cells, and are altered in cancer. These are divided into two groups: oncomirs (on-regulated) that act as oncogenes and anti-oncomirs, which act as tumor suppressors, targeting oncogenes, repressing the cell cycle and cell division in cancer cells. The microRNA *let-7* is an oncogene-anti-oncomir, which regulates the levels of KRAS protein and decreases the rate of cell proliferation (7-9). The rs712 polymorphism that is located in 3′UTR of *KRAS* mRNA is product of one base change of *G* to *T*, and it has been hypothesized that this polymorphism modifies the function of the gene and consequently promotes the cell proliferation and migration in the intestinal mucosa, contributing to CRC carcinogenesis (10). The allele *T* (rs712) showed a frequency of 16% - 36% among controls Chinese population (11). Also, a significant association has been demonstrated between the rs712 polymorphism and different cancers in some studies (12). However, in the Mexican population, these associations remain unknown. Thus, the objective of this investigation was to evaluate the association of the rs712 polymorphism in a *let-7* microRNA-binding *KRAS* gene in Mexicans with CRC.

## Materials and Methods

***Study population***

In this study, DNA samples from 281 healthy volunteer donor and 336 CRC patients with confirmed diagnosis not matched by age and sex, were collected. DNA samples were collected since 2013 to 2017 as part of genetic library of our laboratory, which has been analyzed for other genetic markers polymorphisms (3). DNA samples from parental familial were excluded. Procedures were in accordance with 1964 Helsinki declaration, and the participant in the study signed written informed consent approved by 1305 ethical committee of Centro de Investigación Biomédica de Occidente, Instituto Mexicano del Seguro Social.

***Methods***

**Figure 1:**
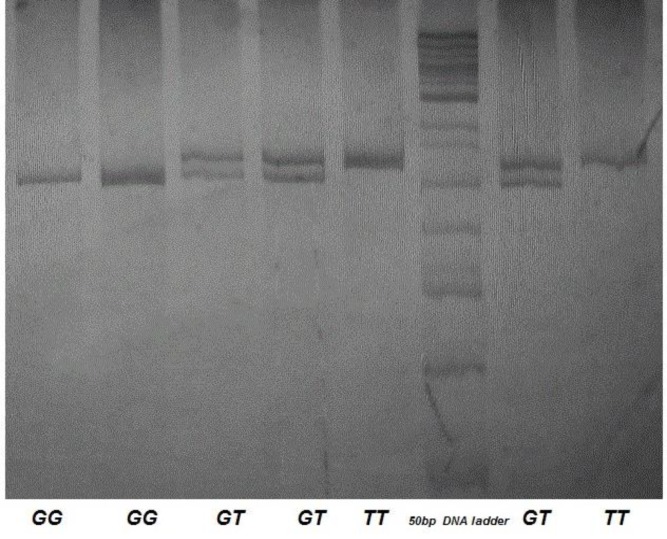
Genotypes representation of the rs712 polymorphism of *KRAS* gene and detection of *GG* [wild genotype, 299 base pairs (bp) and 25 bp bands, digested with *Taq* I restriction enzyme], *TT* (polymorphic genotype, 324 pb band, undigested) and *GT* (heterozygous genotype, 324 bp, 299 bp and 25 bp bands)

DNA sample was obtained by salt precipitation method (13), for the amplification by polymerase chain reaction (PCR). To evaluate the rs712 polymorphism, the following reagents and primers were utilized. The primers: 5′-ATGACAGTGGAAGTTTTTTTTTCCTC-3′ and 5′-GAATCATCATCAGGAAGCCCAT-3′ (14), with 50 ng of genomic DNA, 7.5 pmol of primers, 2.5 mM MgCl_2_, 2.5 U of Taq polymerase (Invitrogen, Carlsbad, CA USA), 0.2 mM dNTPs and 0.1% BSA (Bovine Serum Albumin, New England BioLabs Inc; Beverly, MA, USA) in a 15 ml of total volume. Annealing temperature was 56.5 ^°^C. The allele was identified on 6% polyacrylamide gel by previous enzymatic digestion (*Taq* I; New England BioLabs Inc; Beverly, MA, USA) stained by silver nitrate (15). *GG* genotype (wild type) was identified as the digested fragment and *TT* genotype (polymorphic type) as the undigested fragment (Figure 1).

***Statistical analysis***

Hardy-Weinberg equilibrium (HWE) was tested in the control group. The association analysis was performed by OR using the PASW Statistic Base 18 software, 2009 (Chicago, IL, USA). 

## Results

***Epidemiological data***

Comparative epidemiological data of the studied groups are shown in the Table 1. In CRC patients, the average age was 58.53 (range 30 to 92 years old). Fifty-four percent (180/336) of these patients were male and 77% were ≥50 years old.

***Genotype frequency***

Table 2 shows the genotype distribution of the rs712 polymorphism between CRC patients and controls. The genotype *TT* was observed as protective factor in 18% (59/336) of the patients with CRC and 25% (70/281) of the controls (OR 0.64, 95% CI = 0.43-0.94, *P*=0.0326). The genotype distribution of rs712 polymorphism was in HWE in the control group. Additional differences were observed when the genotypes were analyzed by recessive model (OR 1.55, 95%CI = 1.03-2.34, *P*=0.0326), as a risk factor.

**Table 1:** Demographic data for the study group

	**CRC** ^(n=336)^		**Controls** ^(n=281)^		***P*** **-value**
**Age **(years)					
Mean (SD)*	58.53	(12.49)	40.49	(10.61)	<0.0001
< 50 years [(n), %]	(76)	23.0	(215)	77.0	
≥ 50 years [(n), %]	(260)	77.0	(66)	23.0	<0.0001
**Gender**					
Male [(n), %]	(180)	54.0	(144)	51.0	0.572
Female [(n), %]	(156)	46.0	(137)	49.0	

Colorectal cancer (CRC), SD (standard deviation),

* Student's t-test.

**Table 2:** Genotype and allelic distribution of the rs712 polymorphism of *KRAS* in studied groups

**rs712 polymorphism**		**CRC**		**Controls** *		**OR**	**Confidence intervals (95%)**	***P*** **-value**
Model	*Genotype*	(n=336)	%	(n=281)	%			
	*GG*	(98)	29	(69)	24.5			
	*GT*	(179)	53	(142)	50.5	1.13	(0.82-1.55)	0.4993
	*TT*	(59)	18	(70)	25	0.64	(0.43-0.94)	0.0326
Dominant	*GG*	(98)	29	(69)	24.5			
	*GT/TT*	(238)	71	(212)	75.5	0.79	(0.55-1.13)	0.1991
Recessive	*TT*	(59)	17	(70)	25			
	*GG/GT*	(277)	83	(211)	75	1.55	(1.03-2.34)	0.0326
	*Allele * ^(2n=672)^			^(2n=562)^				
	*G*	(456)	0.6785	(353)	0.6281	1.09	(0.89-1.33)	0.3894
	*T*	(417)	0.3215	(354)	0.3719	0.91	(0.74-1.11)	0.3894

* Controls genotype, Hardy-Weinberg equilibrium in controls group (chi-square test=0.032; *P*=0.8577), Colorectal cancer (CRC).

**Table 3:** Clinical variables of the colorectal cancer and their association with the rs712 polymorphism of KRAS gene

**Clinical variable** *	**Genotype**	**Model**	**Allele**	**OR**	**Confidence intervals (95%)**	***P*** **-value**
Location: colon versus recto			*T*	3.82	(2.77-5.28)	0.0001
			*G*	0.26	(0.18-0.36)	0.0001
Node metastasis: positive	*TT*			2.49	(1.45-4.28)	0.0009
		recessive		0.40	(0.23-0.68)	0.0011
Differentiation: poor	*GT*			2.35	(1.35-4.1)	0.0033
**poor chemotherapy response	*GT*			2.6	(1.7-4.24)	0.0001

* Non-significance clinical variables in analysis included: gender (male, female), age (<50, ≥ 50 years), tobacco and alcohol consumption, stage (I-II, III-IV), metastasis .

** non-response to treatment with pro-drug 5-floururacil (5-FU) and capecitabine was evaluated according to the pathological Ryan's classification described as follows: 1. moderate response (single cells or small groups of cancerous cells), 2. minimum response (residual cancer surrounded by fibrosis), and 3. poor response (minimal or no tumor destruction, extensive residual cancer) (26).

***Comparative analysis with genotypes and clinical characteristics of CRC patients***

Significant differences were found with regards to clinical characteristics of the CRC group and genotypes, alleles and genetics model of the rs712 polymorphism. Colon cancer localization and allele *T* (OR 3.82, 95% CI 2.77-5.28, *P*=0.0001), positive tumor node metastasis with* TT* genotype (OR 2.49, 95% CI 1.45-4.28, *P*=0.0009), poor differentiation (OR 2.35, 95% CI 1.35-4.1, *P*=0.0033) and poor chemotherapy response (OR 2.6, 95% CI 1.7-4.24, *P*=0.0001) with heterozygous (*GT*) genotype, were a risk factor for CRC (Table 3). 

## Discussion

In Mexico, the CRC is a growing morbidity-mortality problem (1-4). Our results do agree with previous reports in the literature, which described an average age of 50 years in individuals with CRC (1-5). Probably, lifestyle changes in terms of diet and changes in longevity have influenced the increased frequency of this disease in the Mexican population (3). 

Knowledge on the molecular mechanisms of colorectal carcinogenesis is major goal; many relevant studies have demonstrated association between CRC and different polymorphisms on *KRAS *gene, such as rs712 polymorphism that has been shown to alter the *let*-7 binding site and regulate KRAS activity affecting gene expression and promoting the cell proliferation in the intestinal mucosa (12, 16). 

Studies have demonstrated an association between polymorphism and an increased severity and susceptibility to various diseases including CRC (11, 12). However, the association of this polymorphism with the cancer depends probably on environmental factors that can promote tumoral epigenetic changes (17). The result observed in this study show that the presence of *TT *variant was a protective factor for CRC. These results were different from a meta-analysis in digestive system cancer of China’s population (11). However, other studies have demonstrated the association with decreased risk for the *T* allele*,* and dominant model *GT/TT* of rs712 polymorphism in breast cancer of Iranian population (18). It has been suggested that *let-7* acts as tumor suppressor that can regulate the expression of distinct pathways required in the tumoral behavior (19). 

Nevertheless, the *T *allele and genotype* TT* were observed as risk factors in patients with colon localization, and nodule metastasis, respectively. As we mentioned, *let-7* has an important participation in the development of metastasis, and the polymorphisms in *Let*-7 modifies binding site that regulates KRAS activity by affecting gene expression and promoting cell proliferation (12).

Other data shows risk association between genotype *GT* of rs712 *let-7* polymorphism in CRC patients with poor differentiation; similar data were found by Jiang *et al* (10) that suggested the possible participation of rs712 in the progression of CRC. 

The association of* GT* genotypes with non-response at adjuvant chemotherapy treatment in patients with CRC [Capecitabine (Xeloda, Roche, oral drug) and pro-drug 5-floururacil (5-FU) are chemotherapeutic drugs commonly used in CRC individuals] was evident. Regarding the rs712 polymorphism in *let-7* region on *KRAS* gene, it could alter the expression mechanism by affecting *let-7* binding region that regulates the constitutive expression of *KRAS* (12). It has been observed that approximately 30% of CRC tumors have mutations in *KRAS* that are predictive of high grade, and aggressive tumors with poor response to chemotherapy, which lead to poor prognosis (20). These mutations alter the GTPase activity of KRAS and could activate proliferative signaling pathways (12. 21). However, the precise mechanism to understanding the therapeutic efficacy of miRNAs is not well-defined. Also, it has been observed that the presence of metastasis is associated with adverse clinical outcome and might alter the expression of different molecular factors included miRNAs such miR-21 and *let-7*, which participate in the regulation of cellular processes (12, 22-24). It has also been observed that the response to drugs not only is related to the monogenic inheritance of a protein variant but also depends on several genes encoding proteins involved in multiple metabolic pathways, posttranslational modifications, gene interactions, and epigenetics (25).

## Conclusion

Our results showed an association of polymorphism as protective factor with CRC when compared with controls. However, this polymorphism could be a good marker in patients for 1) colon localization, 2) node metastasis, 3) poor differentiation, and 4) poor response to chemotherapy factors that could contribute to CRC susceptibility in Mexican population. Similar analyses are necessary to confirm these observed evidences.
